# Vision Based Computing Systems for Healthcare Applications

**DOI:** 10.1155/2019/9581275

**Published:** 2019-02-14

**Authors:** Subrahmanyam Murala, Santosh Kumar Vipparthi, Zahid Akhtar

**Affiliations:** ^1^Department of Electrical Engineering, Indian Institute of Technology Ropar, Roopnagar, India; ^2^Department of Computer Science and Engineering, Malaviya National Institute of Technology, Jaipur, India; ^3^Department of Computer Science, University of Memphis, Memphis, USA

Vision-based systems for healthcare applications utilize computer vision techniques to provide intelligent support to patients and medical practitioners for common needs to sophisticated technical assistance. With the ease of availability and advancements in sensing, mobile devices, and computing power, development of advanced computer vision techniques for assistive healthcare is actively being pursued both by researchers and industries. These systems are built with a combination of pattern recognition, computer vision, machine learning, sensing devices, medical records, expert suggestions, and feedbacks from the medical practitioners. Various vision-based systems are being used in biomedical image analysis, patient monitoring, gesture recognition, gait analysis, advanced rehabilitation, and human psychology, i.e., mental behaviour analysis and lie detection/microexpression analysis.

Pattern recognition is one of the essential parts of the vision-based analytics system. The efficacy of any pattern recognition technique primarily depends on two aspects: feature extraction and classification. As a result, extensive efforts have been made in designing robust, accurate feature descriptors and developing relevant classification techniques. Srensen et al. [1] quantitatively analyzed pulmonary emphysema in computed tomography (CT) images of the lungs using local binary patterns (LBPs). Similarly, Mandal et al. [2] designed ANTithetic Isomeric Cluster Patterns (ANTIC) for robust query-based retrieval of CT images. For analysis of MRI images, numerous variants of LBP have also been proposed in the literature. Vipparthi et al. [3] proposed local directional mask maximum edge patterns for MRI image retrieval. Furthermore, Murala and Wu [4] used local mesh patterns for MRI image indexing and retrieval. The researchers have also used feature descriptors to grade the retinopathy images for diabetic retinopathy detection. The retinopathy grading is specified based on the number of haemorrhages, microaneurysms, and the sign of neovascularization. The absence of these abnormalities constitutes a normal image. Mandal et al. [2] distinguished these features using ANTIC feature descriptors.

Computer vision approaches are also being used for patient behaviour analysis through emotion recognition, gait analysis, and gesture recognition. Various human-computer interaction systems have been developed for improved patient diagnosis and communication between the patient and doctors.

Recent advances in deep learning techniques [5] have led to development of a more intelligent computer-aided detection and analysis system. Ronneberger et al. [6] designed a U-Net convolutional neural network (CNN) for biomedical image segmentation. In addition, Falah et al. [7] employed virtual reality- (VR-) based visualization and training environments in the delivery of anatomy teaching transferring the learning experience.

The scope of visual analytics in healthcare applications is becoming more relevant due to the rise of deep learning techniques, leading to very high accuracy in various computer vision tasks such as image classification, object detection, and semantic segmentation. The potential of these deep networks in medical image analysis has already been tested by the recent work. However, there are many important and interesting challenges such as contextual understanding of visual information and interpretability of deep learning models. We hope that the readers will find interesting research for vision-based healthcare applications in this special issue.
